# Progressive unilateral leg weakness after lumbar decompression due to ischemic monomelic neuropathy misdiagnosed as epidural hematoma: A CARE-compliant case report

**DOI:** 10.1097/MD.0000000000033734

**Published:** 2023-05-12

**Authors:** Gun Woo Lee, Wook-Tae Park, Min Cheol Chang

**Affiliations:** a Department of Orthopedic Surgery, Yeungnam University Medical Center, Yeungnam University College of Medicine, Daegu, Republic of Korea; b Department of Physical Medicine and Rehabilitation, Yeungnam University Medical Center, Yeungnam University College of Medicine, Daegu, Republic of Korea.

**Keywords:** arterial occlusion, ischemic monomelic neuropathy, motor weakness, pain, spinal epidural hematoma

## Abstract

Ischemic monomelic neuropathy (IMN) is a disease that occurs after acute arterial occlusion or steal phenomenon in an extremity that results in single or multiple axonal mononeuropathies in the distal limb without the classical features of limb ischemia, including a skin color change, limb swelling, and ischemic claudication. IMN can easily be misdiagnosed as any other neuropathic disorder. Here we present a case of IMN that was misdiagnosed as spinal epidural hematoma.

**Patient concerns:**

A 77-year-old man presented with sudden motor weakness and pain in his left foot and calf 5 days after a bilateral L4 to 5 posterior decompression for lumbar spinal stenosis. His symptoms progressed over the next 5 days. The strengths of the left ankle dorsiflexors, first toe extensors, and ankle plantar flexors were Medical Research Council 0. On brain and whole-spine magnetic resonance imaging, no specific abnormalities correlated with his symptoms were observed. Computed tomography angiography of the lower extremities revealed segmental occlusion of the left common femoral artery and multifocal severe stenoses in the bilateral anterior and posterior tibial arteries of the left leg. No skin color change or swelling was observed in the left lower extremity.

**Diagnosis:**

Based on his clinical features and imaging findings, he was diagnosed with IMN.

**Intervention:**

The patient underwent thrombectomy of the left femoral artery.

**Outcomes:**

After the treatment, his pain almost completely disappeared.

**Lessons:**

When patients exhibit acute-onset pain in the unilateral limb with or without motor weakness but no correlated abnormality on spinal magnetic resonance imaging or computed tomography, clinicians should consider the possibility of IMN.

## 1. Introduction

Motor weakness is among the most common complaints of patients visiting hospitals or clinics. Because various disorders that occur in neuromuscular structures, including the brain, spinal cord, nerve roots, peripheral nerves, and muscles, can cause motor weakness, clinicians should consider several possible disorders corresponding to the clinical features that each patient shows and discriminate among them to ensure the accurate diagnosis of the cause of motor weakness.^[1–3]^ Acute arterial occlusion in the limbs can also induce motor weakness accompanied by pain, paresthesia, skin color change, and muscle ischemia.^[4]^ Ischemic injury of the nerves supplied by the branches of the occluded artery is responsible for motor weakness of the limb.^[4]^ In rare cases of acute arterial occlusion, ischemic axonal mononeuropathies in the affected limb manifest without significant skin or muscle involvement.^[5]^ This is known as ischemic monomelic neuropathy (IMN).^[5]^

After lumbar spine surgery, motor weakness in the lower extremities occasionally occurs as a postoperative complication. The main causes of motor weakness following lumbar spine surgery are spinal epidural hematoma, nerve root edema, insufficient decompression, and root injury caused by surgical procedures such as improper screw placement for internal fixation.^[6]^ Motor weakness due to lumbar spinal epidural hematoma can be unilateral or bilateral depending on the hematoma size and location.^[7]^ Moreover, the degree of motor weakness caused by spinal epidural hematoma is aggravated over time.^[7–9]^

In patients with motor weakness following lumbar spine surgery, the cause should be accurately diagnosed through physical examination, laboratory studies, and imaging studies, and appropriate treatment should be provided in a timely manner. In the current study, we report a patient with progressive acute motor weakness combined with pain in the unilateral lower extremity due to acute occlusion of the common femoral artery; however, the patient was initially suspected to have spinal epidural hematoma because the motor weakness was initiated 5 days after spinal surgery and progressed.

## 2. Case report

**Figure 1.**
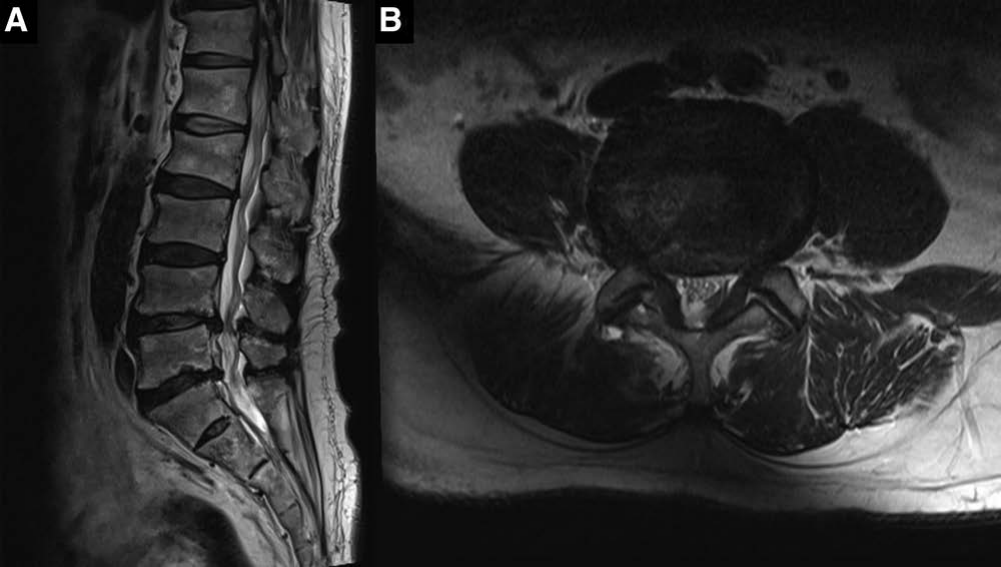
(A) Sagittal and (B) axial T2-weighted magnetic resonance images taken prior to the decompressive laminectomy showing spinal stenosis at the L4 to 5 level.

The current study was approved by the institutional review board of the medical center (YUMC 2023-03-007). A 77-year-old man underwent an L4 to 5 posterior decompression at the department of orthopedic surgery in our university hospital due to persistent lumbar radicular pain in the bilateral posterior thighs and calves (numeric rating scale [NRS, 0: no pain, 10: the worst pain imaginable]: 8, aggravated during waking) for about 5 years due to spinal stenosis, bilateral lateral recess area, on L4 to 5 (Fig. 1). He had a history of diabetes, hypertension, and chronic kidney disease. The patient was taking sitagliptin 25 mg qd and gliclazide 80 mg qd for the treatment of diabetes and furosemide 40 mg bid and nevivolol 2.5 mg qd for the treatment of hypertension. He was undergoing hemodialysis 3 times a week for chronic kidney disease. A physical examination revealed impaired light touch sensation and pain perception at the dermatomes of left L5-S1 and right L5. Motor weakness was not observed preoperatively. The deep tendon reflexes of both lower extremities were decreased. Ankle clonus and flexor plantar reflexes were not present. After a decompressive laminectomy, the pain induced by lumbar spinal stenosis was almost completely relieved and the patient was discharged to the local hospital for postoperative rehabilitation.

**Figure 2.**
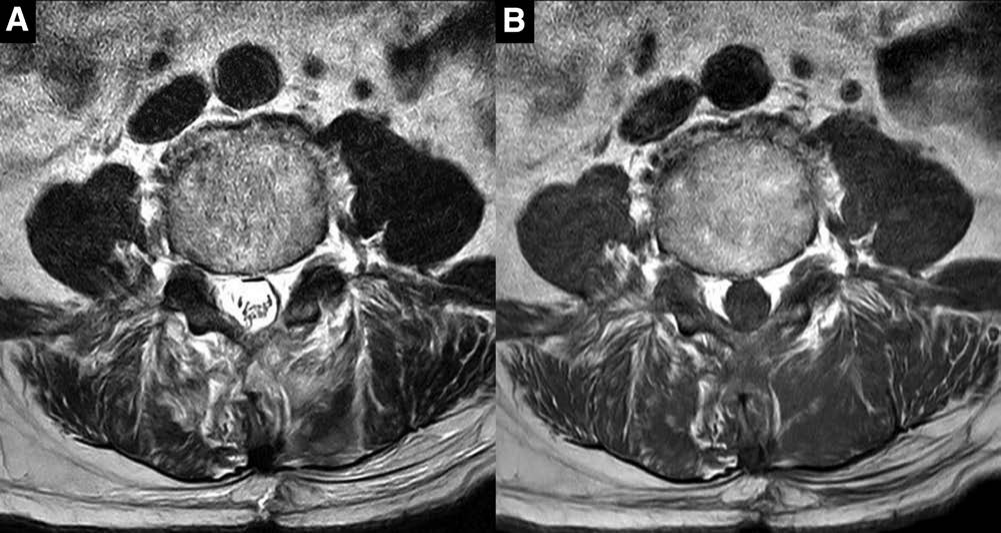
Axial (A) T2-weighted and (B) T1-weighted images taken at 5 days after the decompressive laminectomy showing well-decompressed spinal canal at the L4 to 5 level and no newly developed pathologies.

However, at 5 days postoperative, he visited our hospital’s emergency center for sudden-onset motor weakness and pain in his left foot and calf (NRS: 4). His pain persisted even at rest regardless of changes in body position. The physical examination revealed motor weakness of the left lower extremity. The strengths of the left ankle dorsiflexor, first toe extensor, and ankle plantar flexor were Medical Research Council (MRC) grade 3. Hyperalgesia was also observed in the left L4-S1 dermatome. Decreased deep tendon reflexes were revealed in both lower extremities and ankle clonus and flexor plantar reflexes were lacking. Whole-spine magnetic resonance imaging (MRI) was performed, and the stenotic areas on L4 to 5 level were well-decompressed and no other abnormalities related to the patient’s symptoms were observed (Fig. 2). On the brain MRI, no specific abnormalities were detected. The patient was admitted to the Department of Orthopedic Surgery for evaluation and treatment of the newly developed symptoms.

His symptoms progressed, and at 7 days postoperative, the strengths of the left ankle dorsiflexor, first toe extensor, and ankle plantar flexor were MRC 0, and he could not feel any touch sensations at the left L4-S1 dermatome. No change in skin color or swelling of the lower extremities was observed. Considering the nature of the patient’s symptoms that his motor weakness and pain were initiated at 5 days postoperative, developed at the muscles and areas correlated to the operated lumbar spine level, and progressed over 2 days, a spinal epidural hematoma was highly suspected. Follow-up whole-spine and brain MRI were performed, but no new lesions related to the patient’s symptoms were present. Revision surgery was performed for confirming epidural hematoma and its management if necessary. Perioperatively, no hematoma or compressive lesions were identified; rather, only scar tissue in the epidural and interspinous spaces was noted.

**Figure 3.**
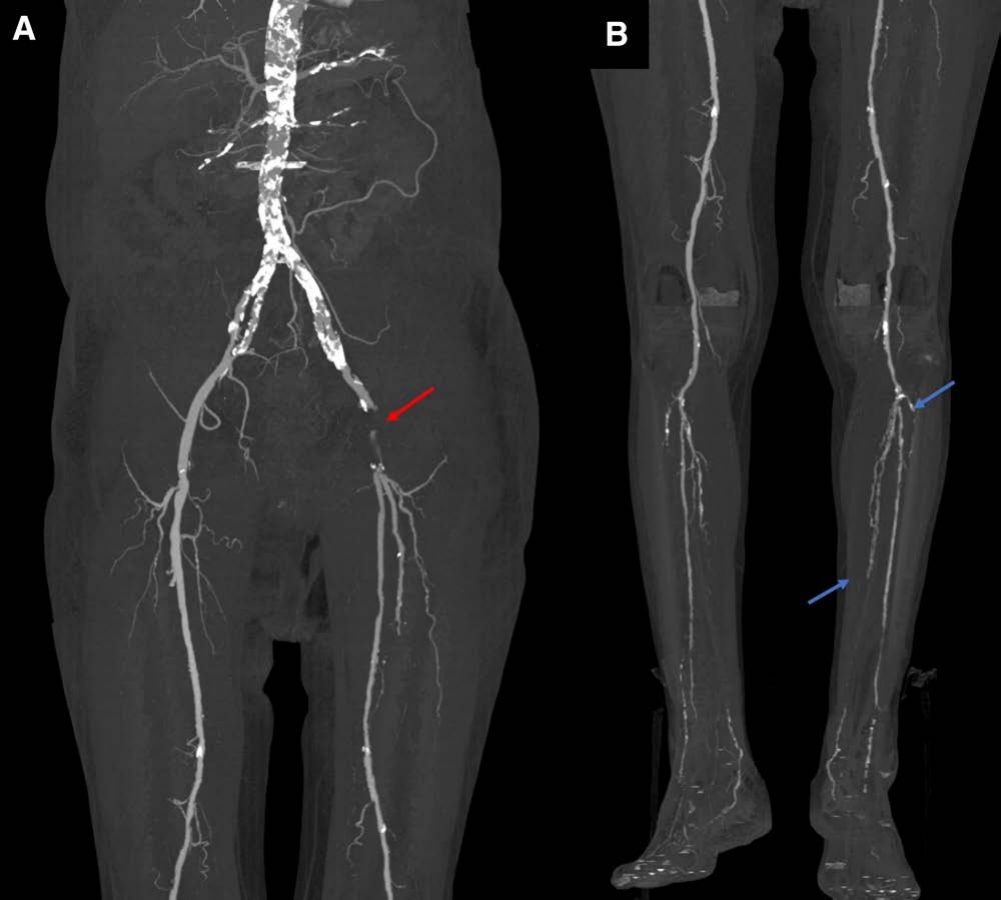
Computed tomography angiography of the lower extremities conducted 14 days after the decompressive laminectomy revealing a left common femoral artery occlusion (red arrow) and multifocal severe stenoses at the bilateral anterior and posterior tibial arteries (blue arrows).

Fourteen days after the initial decompressive surgery, the patient’s motor weakness did not improve, and the pain in the left foot and calf was aggravated (NRS: 7). On the nerve conduction tests, no response was observed in the compound motor action potentials of the left peroneal and tibial nerves recorded at the extensor digitorum brevis and abductor hallucis, respectively. In addition, no sensory nerve action potentials in the bilateral superficial peroneal nerves were noted, and reduced sensory nerve action potentials were observed in the left sural nerve (Rt: 18 μV; Lt: 6 μV). A positive sharp wave (1+) was present in the left tibialis anterior, peroneus longus, medial head of the gastrocnemius, and abductor hallucis muscles. Based on the results of the electrodiagnostic test, the patient was diagnosed with left common peroneal and tibial nerve involvement. In addition, we checked the pulsating of the arteries of the lower extremities. The pulses on the left femoral artery that were checked at the inguinal area and the bilateral dorsalis pedis arteries were weakened. The hand doppler ultrasound confirmed weak pulses of the left femoral, popliteal, anterior tibial, and posterior tibial arteries. Computed tomography (CT) angiography of the lower extremities was performed. Segmental occlusion was noted in the left common femoral artery and multifocal severe stenoses at bilateral anterior and posterior tibial arteries (Fig. 3). Subsequently, the ankle-brachial index was measured in the vascular surgery department; ultimately, 50% stenosis of the left external iliac and thrombosis in the femoral artery were confirmed. We concluded that the patient’s motor weakness was induced by ischemic injury of the left common peroneal and tibial nerves by arterial occlusion. Because our patient’s skin and muscles were not involved, we diagnosed him with IMN. Subsequently, thrombectomy of the left femoral artery, angioplasty with a bovine patch, and balloon angioplasty of the iliac artery were performed. In the subsequent follow-up of CT angiography, normal blood flow was confirmed in the anterior tibial and posterior tibial arteries. In addition, the ankle-brachial index was restored to normal. Aspirin 100 mg 1T qd was administered immediately after thrombectomy, and cilostazol 100 mg 1T qd was started 4 days later. One day postoperative, the patient’s pain had nearly disappeared.

Our patient received rehabilitative treatment (5 days a week, 1 hour/day), including exercises for strengthening the ankle dorsiflexor and plantar flexor muscles and enhancing stability during standing and gait. One month after the thrombectomy with angioplasty, the patient reported no pain in the left lower extremity. However, the strength of the left ankle dorsiflexor, first toe extensor, and ankle plantar flexor did not significantly improve (MRC 1).

## 3. Discussion

Here we reported the case of a patient with severe motor weakness and pain in the left lower extremity due to ischemic injury of the left common peroneal and tibial nerves caused by arterial occlusion and stenosis of the left common femoral, anterior, and posterior tibial arteries without the involvement of the skin and muscle. The patient was diagnosed with IMN.

Ischemic nerve injury due to spontaneous arterial occlusion was first described in 1949 as “ischemic neuritis.” The term IMN was first introduced by Wiburn et al in 1983.^[10]^ IMN is non-compressive occlusion of the blood supply or low blood flow of a major proximal limb artery that results in single or multiple axonal mononeuropathies in the distal limb without compromising the muscle or skin.^[10]^ The common causes of IMN are ischemia due to thromboembolic diseases or diabetic angiopathy and trauma.^[10,11]^ It can also be secondary to vascular surgery or procedures.^[10,11]^ The typical clinical symptoms are acute onset of pain in a distal extremity combined with numbness and paresthesia with or without motor weakness.^[10,11]^ Typically, in patients with IMN, the classical features of limb ischemia, including skin color change, limb swelling, and ischemic claudication, are absent. Accordingly, the diagnosis of IMN is frequently delayed and often misdiagnosed as complex regional pain syndrome or lumbar radiculopathy.^[10]^

Spinal epidural hematoma can occur after spinal surgery or procedures such as interlaminar epidural or caudal steroid injection.^[8,9]^ The clinical symptoms include sudden radicular pain, motor weakness, and sensory deficit in unilateral or bilateral lower extremities.^[8,9]^ Hours to days after their initiation, symptoms insidiously progress. The clinical features of spinal epidural hematoma are similar to those of IMN. Thus, in the case of post-decompressive surgery of the spine, spinal epidural hematoma should be differentiated for the diagnosis of IMN.

Previous studies reported that symptoms induced by IMN are reversible.^[5,10,11]^ Rapid diagnosis and treatment are of particular importance for preventing irreversible nerve injury and maximizing neurological recovery. The delayed diagnosis and treatment of IMN can result in poor outcomes. Therefore, clinicians should be sufficiently aware of its possibility. When patients show acute-onset pain in the unilateral limb with or without motor weakness but no correlated abnormality on spine MRI or CT, the possibility of IMN should be considered.

## Author contributions

**Conceptualization:** Gun Woo Lee, Wook-Tae Park, Min Cheol Chang.

**Data curation:** Gun Woo Lee, Wook-Tae Park, Min Cheol Chang.

**Investigation:** Gun Woo Lee, Wook-Tae Park, Min Cheol Chang.

**Methodology:** Gun Woo Lee, Wook-Tae Park, Min Cheol Chang.

**Resources:** Gun Woo Lee, Wook-Tae Park, Min Cheol Chang.

**Supervision:** Min Cheol Chang.

**Validation:** Gun Woo Lee, Wook-Tae Park, Min Cheol Chang.

**Visualization:** Gun Woo Lee, Wook-Tae Park, Min Cheol Chang.

**Writing – original draft:** Gun Woo Lee, Wook-Tae Park, Min Cheol Chang.

**Writing – review & editing:** Gun Woo Lee, Wook-Tae Park, Min Cheol Chang.
